# 5-Demethoxy-10′-ethoxyexotimarin F, a New Coumarin with MAO-B Inhibitory Potential from *Murraya exotica* L.

**DOI:** 10.3390/molecules27154950

**Published:** 2022-08-03

**Authors:** Zhen-Ru Xia-Hou, Xiao-Fei Feng, Yu-Fei Mei, Yin-Yan Zhang, Tong Yang, Jie Pan, Jing-Hua Yang, Yun-Song Wang

**Affiliations:** 1 Key Laboratory of Medicinal Chemistry for Natural Resource, Ministry of Education, Yunnan Provincial Center for Research & Development of Natural Products, School of Chemical Science and Technology, Yunnan University, Kunming 650091, China; xhzr546552125@163.com (Z.-R.X.-H.); ynu2012@163.com (Y.-F.M.); zyy20220730@163.com (Y.-Y.Z.); yangtongkm@163.com (T.Y.); panjie8668@163.com (J.P.); 2 Faculty of Life Science, Southwest Forestry University, Kunming 650224, China; xiaofei@swfu.edu.cn

**Keywords:** Rutaceae, *Murraya exotica* L., coumarins, MAO-B inhibitor, molecular docking, pharmacophore model

## Abstract

Rutaceae plants are known for being a rich source of coumarins. Preliminary molecular docking showed that there was no significant difference for coumarins in *Clausena* and *Murraya*, both of which had high scoring values and showed good potential inhibitory activity to the MAO-B enzyme. Overall, 32 coumarins were isolated from *Murraya exotica* L., including a new coumarin 5-demethoxy-10′-ethoxyexotimarin F (**1**). Their structures were elucidated on the basis of a comprehensive analysis of 1D and 2D NMR and HRMS spectroscopic data, and the absolute configurations were assigned via a comparison of the specific rotations and the ECD exciton coupling method. The potential of new coumarin (**1**) as a selective inhibitor of MAO-B was initially evaluated through molecular docking and pharmacophore studies. Compound (**1**) showed selectivity for the MAO-B isoenzyme and inhibitory activity in the sub-micromolar range with an IC_50_ value of 153.25 ± 1.58 nM (MAO-B selectivity index > 172).

## 1. Introduction

Depression, Alzheimer’s disease, and Parkinson’s disease, as three major neuropsychiatric diseases, have seriously affected human health and quality of life. Monoamine oxidase (MAO, EC 1.4.3.4) is a flavoenzyme bound to the mitochondrial outer membranes of the cells, which is responsible for the oxidative deamination of neurotransmitters and dietary amines. It exists in two isoforms, MAO-A and MAO-B. Although sharing 70% sequence identity, MAO-A and B displayed different substrate and inhibitor specificities; serotonin and norepinephrine are preferentially metabolized by MAO-A and phenylethylamine, benzylamine, and dopamine by MAO-B, whereas clorgyline and L-deprenyl selectively inhibit MAO-A and B, respectively. They are the well-known target for antidepressants, Parkinson’s disease, and neuroprotective drugs [1,2,3,4]. Monoamine oxidase inhibitors (MAOIs) were used for the treatment of various neurodegenerative disorders. However, they are associated with serious side effects and lack efficacy and selectivity for a single MAO isoform. It is commonly accepted that the discovery of MAOIs from herbal sources is an important strategy for drug design and development to treat various neurodegenerative diseases such as depression, anxiety, Parkinson’s disease, and Alzheimer’s disease [5].

Plant-derived coumarins are an important class of compounds due to their significant biological activities. Coumarins are abundantly found in species of the Rutaceae family, such as the genus *Clausena* and *Murraya* [6,7,8,9,10,11,12]. Our previous studies obtained a novel coumarin, anisucoumaramide, from *Clausena anisum-olens*, which was found to exhibit high selectivity and inhibitory activity in the nanomolar range against MAO-B with an IC_50_ value of 143.65 ± 0.90 nm [6]. Therefore, we hope to continue to obtain novel coumarins with similar activity from Rutaceae plants. *Murraya exotica* (Rutaceae) as a dwarf tree or an evergreen shrub was commonly cultivated in gardens as an ornamental plant in many tropical and subtropical regions. It is an important medicine used for treating fever, cough, and infectious wounds and eliminating pain from injury and trauma [13]. Previous studies have shown that several types of secondary coumarins in *M. exotica* are widely used in the medical, spice, and seasoning industries [8]. In this study, the MAO-B inhibitory potential of coumarins reported from *Clausena* and *Murraya* (Rutaceae) was predicted by molecular docking. The results showed that there was no significant difference between the two. Most of them had certain inhibitory potential, and about one-third of them had better scores. As a continuation of a search for more bioactive coumarins from Rutaceae species, 32 coumarins were isolated from *Murraya exotica* L., including a new coumarin 5-demethoxy-10′-ethoxyexotimarin F (**1**). It was preliminarily found that the new coumarin 1 may have high MAO-B selective inhibitory activity through molecular docking and pharmacophore model study. The determination of hMAO isoform activity displayed (**1**) showed selectivity for the MAO-B isoenzyme and inhibitory activity in the sub-micromolar range with an IC_50_ value of 153.25 ± 1.58 nM (MAO-B selectivity index > 172), of 26.3 ± 1.03 μM to the MAO-A.

## 2. Results and Discussion

### 2.1. Molecular Docking of Coumarins in Clausena and Murraya

Through a previous literature collection, 141 known coumarins were found in *Clausena* plants and 177 coumarins found in *Murraya* plants. Molecular docking studies on coumarins from *Clausena* and *Murraya* were carried out, and the potential inhibitory activity of monoamine oxidase B of the two was compared. The docking results are shown in the following table (Table 1). A negative scoring value indicated that they could bind to the receptor protein. A smaller scoring value indicated better binding [14]. The results showed that only the compounds had better scores, those with scores below −85. The preliminary screening results showed that 40 of the coumarins in *Clausena* had the better scoring value, accounting for 28.4% of the total number, and 48 of the coumarins in *Murraya* had the better scoring value, accounting for 27.1% of the total number. The coumarins of *Murraya* and *Clausena* have similar molecular docking scores; there was no significant difference between the two. Therefore, *Murraya* could be a highly valuable research resource for its coumarins that have MAO-B inhibition potential.

### 2.2. Structure Elucidation of New Coumarin **1**

**Figure 1:**
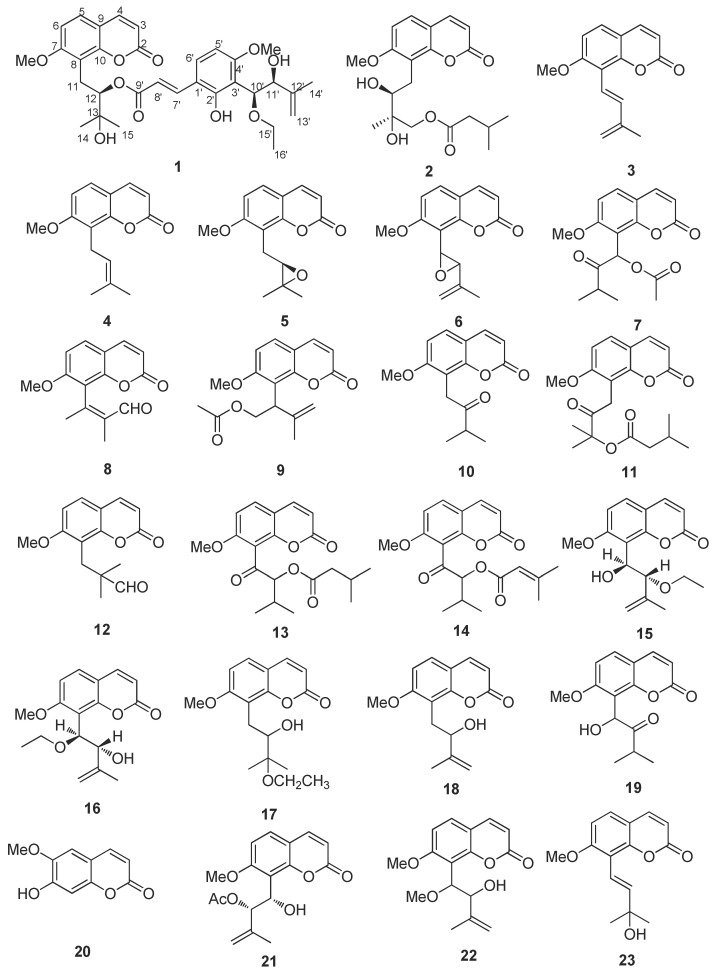

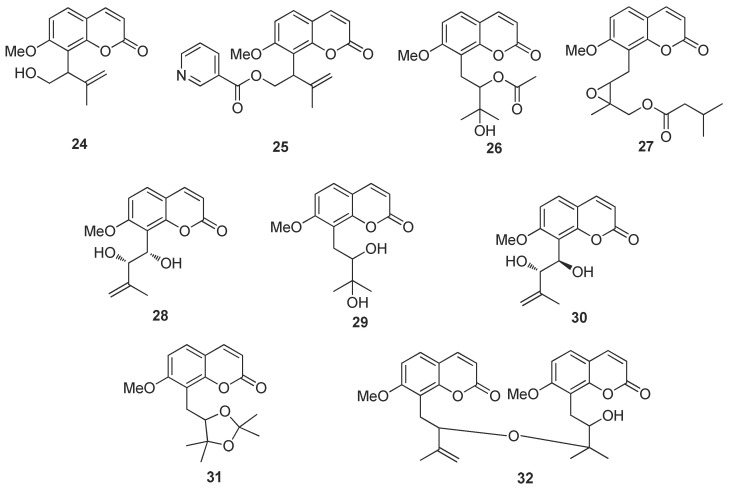
The structures of coumarins from *Murraya exotica* L.

The powdered leaves and twigs of *Murraya exotica* L. (2.5 kg) were repeatedly extracted with 95% EtOH at room temperature. The extract was then concentrated under reduced pressure to give a brown syrup, which was suspended in water and successively partitioned with petroleum ether, ethyl acetate (EtOAc) and *n*-butanol (*n*-BuOH). The EtOAc fraction was subjected to a multi-step chromatography procedure to yield 32 coumarins (Figure 1), including a new coumarin (**1**).

**Figure 2:**
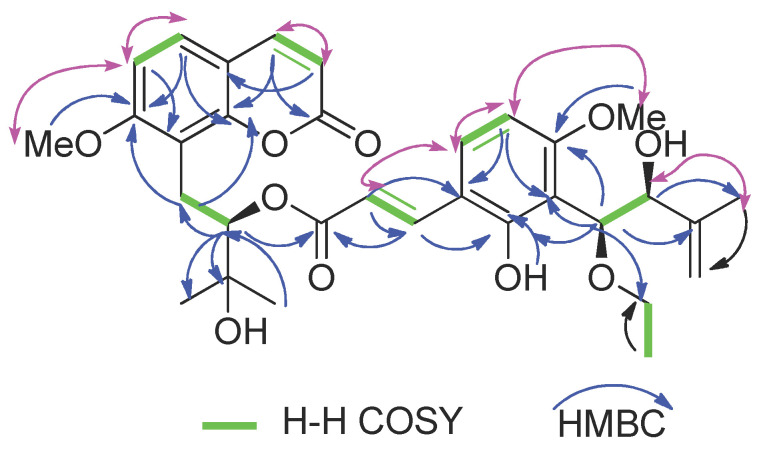
Key ^1^H-^1^H COSY and HMBC of new compound (**1**).

Compound (**1**) was obtained as a white solid. Its molecular formula was determined as C_32_H_38_O_10_ via the positive HRESIMS (*m/z* 605.2359 [M + Na]^+^, calcd for 605.2357) and ^13^C-NMR data, indicating 14 indices of hydrogen deficiency. Strong UV bands at λ_max_ 322 nm showed the characteristic absorption of the coumarin skeleton [15], and the IR spectrum showed absorption bands for carbonyl (1732 cm^−1^) and aromatic ring (1600 cm^−^^1^ and 1465 cm^−1^) functionalities [15]. The ^13^C-NMR, DEPT, and HSQC spectra of (**1**) show the presence of 32 resonances comprised of 4 methyl carbons, 3 methylene carbons, 11 methylene carbons, 12 quaternary carbons and 2 methoxy carbons (see Supplementary Materials). The presence of an 8-prenylated-7-methoxycoumarin backbone as a common structural unit in (**1**) was further deduced by a methoxy singlet signal at *δ* 3.92, two sets of ^1^H AB doublets at *δ*_H_ 6.11 and 7.53 (each d*, J* = 9.4 Hz) and *δ*_H_ 7.29 and 6.79 (each d, *J* = 8.6 Hz), which were easily assignable to H-3 and H-4and to H-5 and H-6 on the coumarin skeleton, respectively, and a group of prenyl signals [*δ*_H_ 3.41 (1H, dd, *J* = 13.6, 10.8 Hz, H-11), 3.13 (1H, dd, *J* = 13.6, 2.4 Hz, H-11), 5.23 (1H, dd, *J* = 10.6, 2.5 Hz, H-12), 1.41 (3H, s, H-14), 1.35 (3H, s, H-15) (Table 2, see Supplementary Materials) [15,16]. The NMR spectroscopic data of (**1**) were similar to those of 10′-ethoxyexotimarin F [16], except that the absence of a methoxy group at C-5, which was deduced from its HMBC correlations of H-5 (*δ*_H_ 7.29) with C-4, C-7, C-9 and C-10 (Figure 2). The ^1^H-NMR spectrum exhibited a group of characteristic signals at *δ*_H_ 7.70, 6.28 (each 1H, d, *J* = 16.1 Hz), and 7.31, 6.38 (each 1H, d, *J* = 8.7 Hz)], confirmed the presence of a set of 2, 3, 4-trisubstituted cinnamoyl unit. 

**Figure 3:**
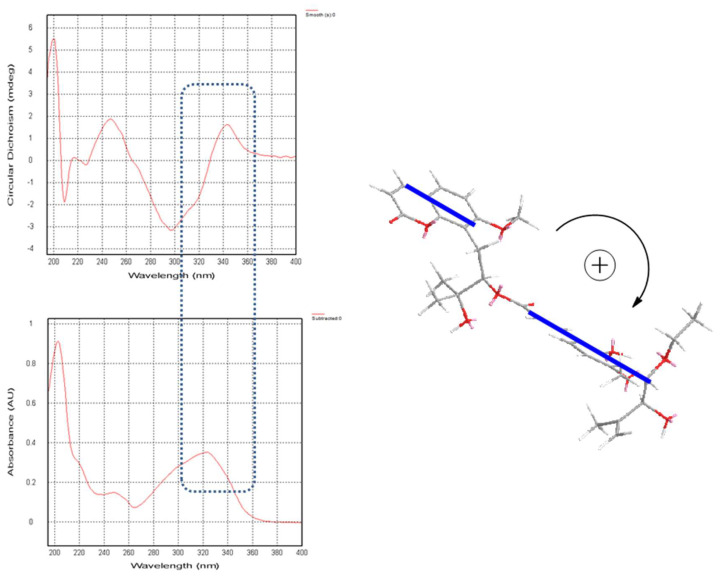
CD spectrum, UV spectrum, and the exciton chirality of **1**.

In (**1**), a large coupling constant (*J* = 7.4 Hz) suggested the *threo* configuration of H-10′/H-11′ [16]. Recently, the circular dichroism exciton chirality method provided a powerful and rapid approach for assigning the absolute configuration of natural products [17]. The exciton chirality method was used to infer the configuration of compound (**1**) based on the sign of the excitonic couplet, using 10′-ethoxyexotimarin F as the reporter group. Thus, the absolute configuration of C-12 was deduced as *R* from a positive split chirality [298 nm (Δε −3.2), 344 nm (Δε +1.6)] determined from the ECD spectrum using the exciton chirality rule (Figure 3). The optical rotation of [α]^26^.^7^_D_ −37.24 (c 0.14, MeOH) of (**1**) with the same negative sign of 10′-ethoxyexotimarin F supposed the same absolute configuration. Moreover, from a biosynthetic consideration, the absolute configurations of (**1**) were deduced to be identical to those of 10′-ethoxyexotimarin F. Thus, the absolute configuration of (**1**) was assigned as (12*R*, 10′*S*, 11′*S*). Hence, compound (**1**) was defined as 5-demethoxy-10′-ethoxyexotimarin F [(*E*)-(*R*)-3-hydroxy-1-(7-methoxy-2-oxo-2*H*-chromen-8-yl)-3-methylbutan-2-yl·3-(3-((1*S*, 2*S*)-1-ethoxy-2-hydroxy-3-methylbut-3-en-1-yl)-2-hydroxy-4-methoxyphenyl)acrylate].

The 31 known coumarins (Figure 1) were identified by comparing their spectroscopic data with those reported in literature as exotimarin G (**2**) [7], *trans*-dehydroosthol (**3**) [7], osthol (**4**) [18], meranzin (**5**) [19], phebalosin (**6**) [19], hainanmurpanin (**7**) [20], murralonginal (**8**) [21], isomurralonginol acetate (**9**) [22], isomeranzin (**10**) [22], paniculonol isovalerate (**11**) [23], 7-methoxy-8-(2-formyl-2-methylpropyl)coumarin (**12**) [24], muralatin O (**13**) [19], isomurranganonsenecioate (**14**) [22], 2′-*O*-ethylmurrangatin (**15**) [25], muralatin K (**16**) [10], yuehgesin-C (**17**) [26], auraptenol (**18**) [22], murranganon (**19**) [22], scopoletin (**20**) [27], murrangatin acetate (**21**) [22], albiflorin-3 (**22**) [28], murraol (**23**) [22], isomurralonginol (**24**) [22], isomurralonginol nicotinate (**25**) [22], 2′-acetoxy-3′-dihydroxyl-osthole (**26**) [29], muralatin P (**27**) [19], murrangatin (**28**) [30], meranzin hydrate (**29**) [15], minumicrolin (**30**) [22], pranferin (**31**) [30], and murradimerin A (**32**) [31].

### 2.3. Virtual Screen of Coumarins Isolate from Murraya exotica L.

**Figure 4:**
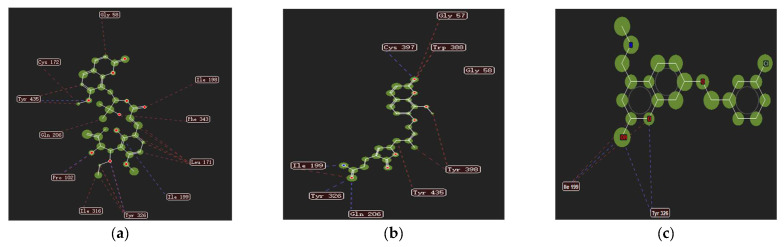
Protein crystals 2D interactions with (**1**), anisucoumaramide, and C18_1503. (**a**) 5-demethoxy-10′-ethoxyexotimarin F (**1**); (**b**) anisucoumaramide; (**c**) C18_1503.

A total of 32 coumarin molecules from *Murraya* *exotica* L. were screened by molecular docking with the ligand C18_1503 in the target protein as the reference and the coumarin anisucoumaramide as the positive control. The results are shown in the following Table 3. The scores showed that nine coumarins were better, accounting for 28.1% of the total number, which was similar to the results in the previous study. In addition, the new coumarin 5-demethoxy-10′-ethoxyexotimarin F (**1**) had a higher scoring value and a higher docking advantage than the positive control anisucoumaramide and the original ligand C18_1503. Therefore, 2D diagrams of the interactions between the new coumarin (**1**), anisucoumaramide and C18_1503 and protein crystals were displayed, respectively (Figure 4). It could be found that all three had hydrogen bond interaction, spatial interaction with residue Ile 199, and hydrogen bond interaction with residue Tyr 326, which was consistent with the action reported in the literature [32,33]. Therefore, the new compound 5-demethoxy-10′-ethoxyexotimarin F (**1**) had a certain selective inhibition potential for MAO-B.

In the pharmacophore study of MAO-B selective inhibitor, the pharmacophore model **01** had the highest CAI value (see Table 4). Moreover, its other indicators were also high. Therefore, the pharmacophore model **01** was determined as that optimal pharmacophore. According to the obtained optimal pharmacophore, 32 coumarins isolated from *Murraya exotica* L. were virtually screened. The screening results are shown in Table 5. Overall, 27 molecules could be matched with the pharmacophore, indicating that the coumarins in *Murraya exotica* L. had high overall matching with the pharmacophore model of MAO-B selective inhibitor. The matching of each molecule was evaluated based on the Fit Value. A higher value indicated a higher degree of matching between the molecule and the pharmacophore model. As can be seen from the table, the new compound 5-demethoxy-10′-ethoxyexotimarin F (**1**) has a high matching property and, therefore, a high potential as a selective inhibitor of MAO-B.

### 2.4. Biological Activity of Coumarin **1**

The potential inhibitory effects of the new coumarin (**1**) were evaluated on human recombinant monoamine oxidase (hMAO) isoforms. The inhibitory effects were assessed by measuring the production of H_2_O_2_ from *p*-tyramine using the Amplex Red MAO assay kit with selegiline and iproniazide as reference drugs. The IC_50_ values and MAO-B selectivity indices for the inhibitory effects of both the new compound and reference inhibitors were calculated (Table 6). Compound (**1**) inhibited MAO-B with an IC_50_ value of 153.25 ± 1.58 nM (MAO-B selectivity index > 172) but with an IC_50_ value of 26.3 ± 1.03 μM to the MAO-A, demonstrating that compound (**1**) shows selectivity for the MAO-B isoenzyme and inhibitory activity in the sub-micromolar range.

The anti-inflammatory activity of (**1**) was evaluated by measuring the inhibition against LPS-induced NO production in RAW264.7 cells. Compound (**1**) displayed no inhibitory effect, showing only a 34.59% inhibition, less than 50%, compared with NG-monomethyl-l-arginine (l-NMMA) with an inhibition rate of 56.13%. Meanwhile, the acetylcholinesterase (AChE) and butyrylcholinesterase (BuChE) inhibitory activity of compound (**1**) was assayed. (**1**) did not show any inhibitory activity at a concentration of 50 μM. Tacrine (0.33 μM) was used as the positive control and showed 50.1% inhibition. The α-glucosidase enzymatic activity of compound (**1**) was investigated and found to be inactive since its percentage of inhibition was less than 50% at a concentration of 50 μM compared to 53.17 % inhibition of quercetin (positive control). Compound (**1**) did not lead to a significant inhibitory effect against four bacterial pathogens*, Salmonella enterica* (ATCC14028), *Staphylococcus aureus* (ATCC29213), *Escherichia coli* (ATCC25922), and *Pseudomonas aeruginosa* (ATCC27853), and fungal pathogen *Candida albicans* (ATCC10231) at a concentration of 100 μM.

## 3. Materials and Methods

### 3.1. General Experimental Procedures

The specific optical rotation data were acquired on a Rudolph Autopol III automatic polarimeter (Rudolph Research, Fairfield, NJ, USA). The UV spectra were recorded on a Shimadzu UV-2450 spectrophotometer (Shimadzu Corporation, Kyoto, Japan). IR spectra were recorded on a Thermo Nicolet Nexus 470 FT-IR spectrometer (Thermo Nicolet, Vernon Hills, IL, USA). The ECD data were acquired on a JASCO 810 CD spectrophotometer (Jasco Corporation, Tokyo, Japan). NMR spectra were recorded on a Brucker-400 and 600 NMR spectrometer (Bruker Corp. Billerica, MA, USA), with tetramethylsilane as an internal standard. HRESIMS experiments were measured on a Waters Xevo G2 Q-TOF mass spectrometer (Waters MS Technologies, Manchester, UK). Silica gel (100–200 mesh or 200–300 mesh, Qingdao Marine Chemical Co. Ltd., Qingdao, China) and Sephadex LH-20 (Amersham Biosciences, Uppsala, Sweden) were used for open CC. TLC analyses were carried out on the pre-coated silica gel GF254 plates (Qingdao Marine Chemical Co. Ltd., Qingdao, China). The spots were visualized under the UV lights (254 nm and 365 nm). All of the solvents were distilled prior to use.

### 3.2. Plant Materials

The leaves and twigs of *Murraya exotica* L. were collected in Hekou County, Honghe Prefecture, Yunnan Province in May 2019 and identified by Professor Chen Yu. The sample was deposited in the School of Chemical Science and Technology, Yunnan University.

### 3.3. Extraction and Isolation

Dried powdered leaves and twigs of *Murraya exotica* L. (2.5 kg) were extracted three times with 95% aqueous EtOH. The extract was evaporated under reduced pressure, and the residue (500 g) was suspended in H_2_O and partitioned successively with petroleum ether, EtOAc, and n-BuOH. The EtOAc extract (100 g) was subjected to silica gel CC and eluted with a stepwise gradient of petroleum ether-EtOAc (7:3, 3:2, 2:3, 1:4, and 0:1, *v/v*) to afford five fractions (F1–5), respectively. Then each part was subjected to a series of chromatographic techniques, such as silica gel column (mesh 200–300) and Sephadex LH-20. Fraction 1 (25 g) was separated on silica gel CC eluting with petroleum ether-EtOAc (4:1, *v/v*) and Sephadex LH-20 (CH_2_Cl_2_-MeOH, 1:1, *v/v*) to afford (**3**) (15.1 mg), (**4**) (3.5 mg), (**5**) (5.2 mg), (**6**) (10.1 mg), (**7**) (99.5 mg), (**8**) (11.4 mg), (**9**) (36 mg), (**10**) (16.0 mg), (**11**) (4.5 mg), (**12**) (5.5 mg). Fraction 2 (20 g) was chromatographed successively on silica gel CC and Sephadex LH-20 (CH_2_Cl_2_-MeOH, 1:1, *v/v*) to afford (**13**) (5.5 mg), (**14**) (10.3 mg), (**15**) (18 mg), (**16**) (5.4 mg), (**17**) (25.5 mg), (**18**) (7.7 mg), (**19**) (19.0 mg), (**20**) (7.1 mg). Fraction 3 (25 g) was chromatographed successively on silica gel CC and Sephadex LH-20 (CH_2_Cl_2_-MeOH, 1:1, *v/v*) to afford (**1**) (3 mg), (**2**) (5.3 mg), (**21**) (9.2 mg), (**22**) (1.5 mg), (**23**) (9.5 mg), (**24**) (5.0 mg), (**25**) (9.2 mg), (**26**) (3.1 mg), (**27**) (6.4 mg), (**28**) (18.0 mg), (**29**) (4.5 mg), (**32**) (12.1 mg). Fraction 4 (14 g) was chromatographed on silica gel CC eluting with petroleum ether-acetone (3:2, *v/v*) and Sephadex LH-20 (MeOH) to afford (**30**) (5 mg). Fraction 5 (16 g) was chromatographed on silica gel CC eluting with CH_2_Cl_2_-acetone (4:1, *v/v*) and Sephadex LH-20 (MeOH) to afford (**31**) (51.0 mg).

### 3.4. Structural Elucidation

5-demethoxy-10′-ethoxyexotimarin F (**1**): White solid; [α]^2^^6^.^7^_D_ -37.24 (c0.14, MeOH); UV (MeOH) λ_max_198, 246 and 322 nm; IR (KBr) ν_max_ 3380, 2925, 2855, 1732, 1724, 1600, 1465 cm^−1^; ^1^H and ^13^C NMR data see Table 2; HRESIMS *m*/*z* 605.2359 [M + Na]^+^ (calcd for C_32_H_37_O_10_, 605.2357).

### 3.5. Molecular Docking

Molegro Virtual Docker 6.0 (MVD 6.0, 2013, Molexus, Odder, Denmark) semi-flexible docking software is mainly used, which is higher than the docking accuracy of most software on the market. The key is that it comes with a number of different scoring functions and search algorithms. The combination of the two can obtain the docking algorithm for the receptor protein. After repeated attempts using different scoring functions and search algorithms in the software, a docking method with good repeatability was finally obtained [34]. The RMSD value when reproducing the original protein-ligand was 1.066, less than 2, indicating that the docking method was available. In the finally determined docking method, the PLANTS Score [GRID] is used for the scoring function, and Iterated Simplex is used for the search algorithm. In this study [35], the protein crystal 2V61 obtained from the PDB database was combined with the selective inhibitor 7-(3-chlorobenzyloxy)-4-(methylamino) methyl-coumarin, providing more reference value for the docking analysis of coumarin compounds [36].

### 3.6. Pharmacophore Model

**Figure 5:**
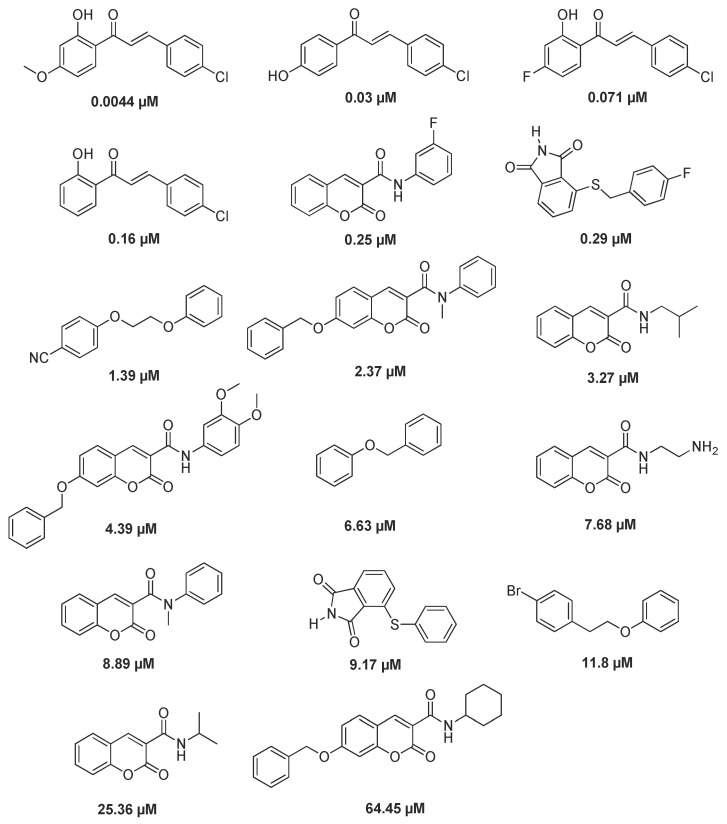
Training set compounds and their activity value.

The pharmacophore model was constructed based on the common pharmacodynamic characteristics [37], and the active compounds related to MAO-B selective inhibitor were obtained by using the protein code P27338 of protein crystal 2V61 and the Binding Data Base database. The training set consisted of 17 active compounds. The specific structure and IC_50_ activity values are shown in Figure 5. The test set consisted of 50 active compounds and 100 inactive compounds [38,39,40,41]. Pharmacophore model evaluation is based on the CAI system, and the evaluation results are shown in Table 4 [42,43,44].

### 3.7. In Vitro Bioassay

#### 3.7.1. In Vitro MAO Inhibitory Assay

The effects of (**1**) on the hMAO isoform enzymatic activity were evaluated by measuring the effects on the production of H_2_O_2_ from *p*-tyramine using a fluorimetric method following the experimental protocol previously described [6]. Selegiline and iproniazide served as reference inhibitors.

#### 3.7.2. Anti-Inflammatory Assay

The Nitric oxide (NO) production inhibition of the compounds (**1**) was determined using a procedure described before [45].

#### 3.7.3. Acetylcholinesterase/Butrylcholinesterase Inhibitory Activity

Acetylcholinesterase/butyrylcholinesterase (AChE/BuChE) inhibitory activity of the isolated compound (**1**) was assayed by the spectrophotometric method developed by Ellman’s method with slight modification [46]. *S*-Acetylthiocholine iodide, *S*-butyrylthiocholineiodide, 5,5′-dithio-bis-(2-nitrobenzoic) acid (DTNB, Ellman’s reagent), acetylcholinesterase and butyrylcholinesterase derived from human erythrocytes were purchased from Sigma Chemical (St. Louis, MO, USA). The compounds were dissolved in DMSO. The reaction mixture (totally 200 μL) containing phosphate buffer (pH 8.0), test compound (50 μM), and acetyl cholinesterase (0.02 U/mL) or butyrylcholinesterase (0.016 U/mL), was incubated for 20 min (37 °C). Then, the reaction was initiated by the addition of 40 μL of the solution containing DTNB (0.625 mM) and acetylthiocholine iodide (0.625 mM) or butyrylthiocholine iodide (0.625 mM) for AChE or BuChE inhibitory activity assay, respectively. The hydrolysis of acetylthiocholineorbutyrylthiocholine was monitored at 405 nm every 30 s for one hour. Tacrine was used as a positive control with a final concentration of 0.333 μM. All of the reactions were performed in triplicate. The percentage inhibition was calculated as follows: % inhibition = (E − S)/E × 100 (E is the activity of the enzyme without test compound, and S is the activity of the enzyme with test compound).

#### 3.7.4. α-Glucosidase Inhibition Assay

An enzyme inhibitor screening model was chosen using 4-nitro-phenol-α-d-glucopyranoside (4-NPGP) and slightly modified [47]. Briefly, the test compound (50 μM), α-glucosidase solution (0.025 U/mL), phosphate buffer (pH 6.9), and (4-NPGP) (1 μM) were incubated in 96-well plates at 37 °C for 50 min. Absorbance at 405 nm was recorded on a microplate reader. Blank readings (no enzyme) were subtracted from each well, and the results were compared to the control. Quercetin was selected as the positive control. All of the reactions were repeated three times. The inhibition rate (%) was calculated as (1 − OD_sample_)/OD_control blank_ × 100%.

#### 3.7.5. Antimicrobial Assay

The broth dilution method and Oxford cup method were used to detect antimicrobial activity against reference strains, including *Salmonella enterica subsp. enterica* (ATCC14028), *Staphylococcus aureus subsp. aureus* (ATCC29213), *Escherichia coli* (ATCC25922), *Pseudomonas aeruginosa* (ATCC27853), and *Candida albicans* (ATCC10231). Ceftazidime and penicillin *G* sodium and amphotericin B served as an antibacterial and antifungal positive control, respectively. The percentage inhibition of cell growth below 50% was regarded as inactive.

## 4. Conclusions

A new coumarin 5-demethoxy-10′-ethoxyexotimarin F (**1**) was obtained from *Murraya exotica* L. (**1**) could be a new potential MAO-B selective inhibitor, which showed better than the known positive control coumarin anisucoumaramide and the original ligand reference C18_1503 through molecular docking and pharmacophore model evaluation. Compound (**1**) showed selectivity for the MAO-B isoenzyme and inhibitory activity in the sub-micromolar range with an IC_50_ value of 153.25 ± 1.58 nM (MAO-B selectivity index > 172), of 26.3 ± 1.03 μM to the MAO-A. The exploration and discovery of bioactive components from medicinal herbs is one of the most important approaches for developing new drugs and improving the therapeutic properties in drug discovery. This study enriches the chemical diversity of coumarins in *Murraya* species and provides a theoretical basis for the traditional usage of *Murraya exotica* L. The results encourage us to further explore the potential of this family of derivatives as potential lead candidates for the treatment of neurodegenerative disorders.

## Figures and Tables

**Table 1:** Docking results of coumarins in *Clausena* and *Murraya*.

*Clausena*		*Murraya*	
Compound	Score	Compound	Score
clauslactone V	−149.541	murradimerin A	−138.086
2′,3′-epoxyamsolactone	−136.470	omphamurin isovalerate	−126.578
clauslactone J	−134.113	murratin L	−121.957
anisocoumarin J	−132.726	panitin C	−119.568
excavacoumarin C	−131.813	C18_1503	−118.516
clauslactone I	−129.619	murratin D	−118.495
clauslactone L	−128.073	6-(2′,3′-dihydroxy-3-methylbutyl)-8-prenylumbelliferone	−116.788
clauslactone F	−127.125	muralatin B	−114.919
anisolactone	−125.489	aurapten	−111.870
clausenalansimin B	−125.462	7-(3-methyl-2-butenyloxy)-8(3-butenyl-3-methyl-2-oxo)-coumarin	−109.601
clauslactone A	−125.350	5-methoxypanial	−108.623
clauslactone S	−125.222	6-methoxy-7-geranyloxycoumarin	−106.905
clauslactone R	−123.325	murratin M	−106.550
clauslactone K	−119.830	isomurralonginol nicotinate	−105.839
C18_1503	−118.516	murralonginol isovalerate	−105.761
clauslactone O	−117.426	mexoticin	−104.093
5-geranyloxy-7-hydroxycoumarin	−117.172	isomurranganonsenecioate	−103.535
hekumarin A	−116.313	murpanicin	−102.054
excavatin B	−116.078	scopolin	−101.550
anisucoumaramide	−115.707	paniculin	−99.904
indicolactone	−113.647	isogosferol	−99.570
excavacoumarin F	−111.570	omphamurin	−98.948
clauslactone C	−108.884	isomexoticin	−97.931
excavacoumarin E	−108.804	murralongic acid	−97.607
phellopterin	−108.137	murpaniculol	−97.455
clauslactone D	−102.058	isomeranzin	−95.308
excavatin M	−101.192	braylin	−94.309
5-hydroxy-8-(3′-methyl-2′-butenyl) furocoumarin	−100.497	yuehgesin-B	−93.991
clausindine	−97.617	murratin G	−93.762
clauslactone W	−96.696	muralatin L	−93.649
(2″*S*)-isosaxalin	−95.188	7-methoxy-8-(5-(prop-1-en-2-yloxy) penta-1,3-dien-1-yl)-coumarin	−93.252
(+)-elisin	−94.884	osthenon	−92.408
peucedanone	−94.700	murraculatin	−92.231
7-methoxy-6-(2′*R*- methoxy-3′-hydroxy-3′-methylbutyl) coumarin	−93.072	muralatin P	−91.867
anisocoumarin F	−92.112	6-hydroxycoumurrayin	−91.595
chalepensin	−91.600	panitin G	−91.252
xanthyletin	−91.432	columbianetin acetate	−91.158
(+)-*trans*-decursidinol	−90.820	minumicrolin	−90.401
heraclenol	−89.300	auraptenol	−89.986
xanthoxyletin	−86.778	5,7-dimethoxy-8-(3′-methyl-2′-oxobutyl) coumarin	−89.605
excavatin F	−86.1468	8-(2′-oxo-3′-methyl)butoxy-7-methoxycoumarin	−89.181
		murratin I	−88.635
		murraxocin	−88.498
		(±)-murratin A	−88.132
		muralatin C	−87.348
		phebalosin	−86.790
		10-methoxy-7-methyl-2*H*-benzo[*g*]chromen-2-one	−86.436
		murratin F	−85.589
		byakangelicin	−85.426

**Table 2:** ^1^H (400 MHz) and ^13^C (100 MHz) NMR data of (**1**) in CDCl_3_.

Position	*δ*_H_ (*J* in Hz)	δ_C_ (Type)	COSY	HMBC
2	/	161.2, C		
3	6.11, d (9.4)	112.8, CH	H-4	C-9
4	7.53, d (9.4)	143.7, CH	H-3	C-2, C-10
5	7.29, d (8.6)	127.1, CH	H-6	C-4, C-7, C-9, C-10
6	6.79, d (8.6)	107.2, CH	H-5	C-7, C-8
7	/	160.8, C		
8	/	114.7, C		
9	/	153.5, C		
10	/	110.3, C		
11a	3.41, dd (13.6, 10.8)	23.3, CH_2_	H-12	C-7, C-8, C-10
11b	3.13, dd (13.6, 2.4)			
12	5.23, dd (10.6, 2.5)	78.7, CH	H-11	C-8, C-11, C-14, C-15, C-9′
13		72.7, C		
14	1.41, s	25.3, CH_3_		C-12, C-15
15	1.35, s	26.6, CH_3_		C-12, C-14
1′	/	116.0, C		
2′	/	156.8, C		
3′	/	110.3, C		
4′	/	160.0, C		
5′	6.38, d (8.7)	102.4, CH	H-6′	C-1′, C-3′
6′	7.31, d (8.7)	130.0, CH	H-5′	C-2′, C-4′, C-7′
7′	7.70, d (16.1)	140.1, CH	H-8′	C-2′, C-6′, C-9′
8′	6.28, d (16.1)	115.8, CH	H-7′	C-1′
9′	/	167.2, C		
10′	4.94, d (7.4)	79.3, CH		C-2′, C-3′, C-4’, C-11′, C-12′, C-15′
11′	4.27, d (7.4)	77.1, CH		C-10′, C-12′, C-13′, C-14′
12′	/	142.9, C		
13′a	4.75, s	113.6, CH_2_		C-11′, C-12′, C-14′
13′b	4.62, s			C-11′, C-12′, C-14′
14′	1.75, s	17.9, CH_3_		C-11′, C-13′
15′a	3.68, dq (14.0, 7.0)	66.5, CH_2_	H-16′	C-10′, C-16′
15′b	3.59, dq (14.0, 7.0)			
16′	1.27, t (7.0)	15.0, CH_3_	H-15′	C-15′
7-OCH_3_	3.92, s	56.2, CH_3_		C-7
4′-OCH_3_	3.75, s	55.4, CH_3_		C-4′
2′-OH	8.98, s			C-2′

**Table 3:** Docking scores of coumarins of *Murraya exotica* L.

Compound	Score
murradimerin A (**32**)	−138.086
5-demethoxy-10′-ethoxyexotimarin F (**1**)	−129.482
C18_1503	−118.516
anisucoumaramide	−115.707
isomurralonginol nicotinate (**25**)	−105.839
isomurranganonsenecioate (**14**)	−103.535
7-methoxy-8-(2-formyl-2-methylpropyl)coumarin (**12**)	−99.9048
Isomeranzin (**10**)	−95.3081
muralatinP (**27**)	−91.8677
minumicrolin (**30**)	−90.4016
phebalosin (**6**)	−86.7904

**Table 4:** Evaluation results of pharmacophore model *.

Model	*H_t_*	*H_a_*	*A*%	*Y*%	*N*	CAI
**01**	44	40	80	90.9	2.73	2.18
**02**	43	39	78	90.7	2.72	2.12
**03**	43	39	78	90.7	2.72	2.12
**04**	48	41	82	85.4	2.56	2.10
**05**	48	40	80	83.3	2.50	2.00
**06**	54	40	80	74.1	2.22	1.78
**07**	54	40	80	74.1	2.22	1.78
**08**	51	40	80	78.4	2.35	1.88
**09**	52	40	80	76.9	2.31	1.85
**10**	66	40	80	60.6	1.82	1.45

* Among them, the larger the CAI value is, the more reliable the pharmacophore model will be explained to some extent. The CAI system is mainly based on the four parameters A%, Y%, N and CAI. H_t_ was the total number of compounds hit by the pharmacophore model, and H_a_ was the number of active compounds hit by the pharmacophore model. A% indicated the hit rate of active components, Y% indicated the effective hit rate, N indicated the effective identification index, and CAI indicated the comprehensive evaluation index.

**Table 5:** Pharmacophore model screening results.

Compound	Fit Value
5-demethoxy-10′-ethoxyexotimarin F (**1**)	2.90864
exotimarin G (**2**)	2.76624
isomurralonginol nicotinate (**25**)	2.73866
muralatin P (**27**)	2.63347
muralatin O (**13**)	2.61354
murradimerin A (**32**)	2.61125
isomurranganonsenecioate (**14**)	2.50786
yuehgesin-C (**17**)	2.49509
paniculonol isovalerate (**11**)	2.16367
2′-*O*-ethylmurrangatin (**15**)	1.8576
muralatin K (**16**)	1.70339
hainanmurpanin (**7**)	1.51473
auraptenol (**18**)	1.23251
pranferin (**31**)	1.2031
murranganon (**19**)	1.14868
isomeranzin (**10**)	0.912292
murrangatin (**28**)	0.79533
murrangatin acetate (**21**)	0.734945
minumicrolin (**30**)	0.725833
meranzin (**5**)	0.536345
albiflorin-3 (**22**)	0.476371
osthol (**4**)	0.316498
isomurralonginol (**24**)	0.265571
phebalosin (**6**)	0.244483
isomurralonginol acetate (**9**)	0.209117
*trans*-dehydroosthol (**3**)	0.104663
7-methoxy-8-(2-formyl-2-methylpropyl)coumarin (**12**)	0.0957507

**Table 6:** MAO-A and MAO-B inhibitory activity results.

Compounds	MAO-A IC_50_	MAO-B IC_50_	SI
(**1**)	26.3 ± 1.03 μM	153.25 ± 1.58 nM	172
selegiline	67.24 ± 1.03 μM	19.58 ± 0.83 nM	3434
iproniazide	6.55 ± 0.75 μM	7.52 ± 0.34 μM	0.87

Each IC_50_ value is the mean ± S.E.M. from three experiments. SI: MAO-B selectivity index = IC_50_ (MAO-A)/IC_50_ (MAO-B).

## Data Availability

The data presented in this study are available on request from the corresponding author.

## Supplementary Materials

The following supporting information can be downloaded at: https://www.mdpi.com/article/10.3390/molecules27154950/s1, Figure S1. ^1^H NMR spectrum of **1** in CDCl3; Figure S2. ^13^C NMR and DEPT spectra of **1** in CDCl3; Figure S3. HSQC spectrum of **1** in CDCl3; Figure S4. HMBC spectrum of **1** in CDCl3; Figure S5. ^1^H-^1^H COSY spectrum of **1** in CDCl3; Figure S6. ROESY spectrum of **1** in CDCl3; Figure S7. HRESIMS spectrum of**1**; Figure S8. UV spectrum of **1**; Figure S9. CD spectrum of **1**.

## Sample Availability

Samples of the compounds are not available from the authors.
