# Supplementary material for: 5-Demethoxy-10′-ethoxyexotimarin F, a New Coumarin with MAO-B Inhibitory Potential from Murraya exotica L
Source: Molecules. 2022 Aug 3;27(15):4950. doi: 10.3390/molecules27154950 (PMC9370560; doi:10.3390/molecules27154950)
Supplement: Supplementary file 1 [file molecules-27-04950-s001.zip › molecules-1785470-supplementary.pdf]

molecules

MDPI

Supplementary Materials

5-Demethoxy-10′-ethoxyexotimarin F, a New Coumarin with MAO-B Inhibitory Potential from *Murraya exotica* L.

Zhen-Ru Xia-Hou [1,†], Xiao-Fei Feng [2,†], Yu-Fei Mei [1], Yin-Yan Zhang [1], Tong Yang [1], Jie Pan [1], Jing-Hua Yang [1,*] and Yun-Song Wang [1,*]

[1] Key Laboratory of Medicinal Chemistry for Natural Resource, Ministry of Education, Yunnan Provincial Center for Research & Development of Natural Products, School of Chemical Science and Technology, Yunnan University, Kunming 650091, China; xhzr546552125@163.com (Z.-R.X.-H.); ynu2012@163.com (Y.-F.M.); zyy20220730@163.com (Y.-Y.Z.); yangtongkm@163.com (T.Y.); panjie8668@163.com (J.P.)

[2] Faculty of Life Science, Southwest Forestry University, Kunming 650224, China; xiaofei@swfu.edu.cn (X.-F.F.)

* Correspondence: yangjh@ynu.edu.cn (J.-H.Y.); wangys@ynu.edu.cn (Y.-S.W.)

† These authors contributed equally to this work.

List of Figures

Figure S1. ^{1}H NMR spectrum of **1** in $CDCl_3$

Figure S2. ^{13}C NMR and DEPT spectra of **1** in $CDCl_3$

Figure S3. HSQC spectrum of **1** in $CDCl_3$

Figure S4. HMBC spectrum of **1** in $CDCl_3$

Figure S5. ^{1}H-^{1}H COSY spectrum of **1** in $CDCl_3$

Figure S6. ROESY spectrum of **1** in $CDCl_3$

Figure S7. HRESIMS spectrum of **1**

Figure S8. UV spectrum of **1**

Figure S9. CD spectrum of **1**

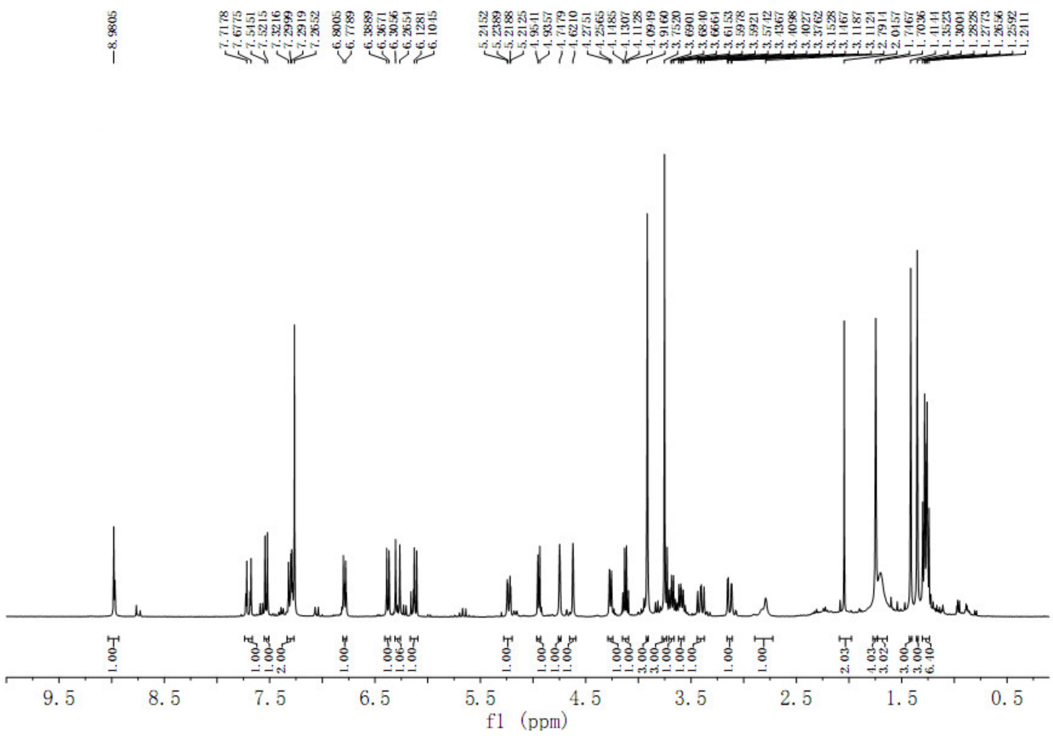

Figure S1. ^{1}H NMR spectrum of **1** in $CDCl_3$

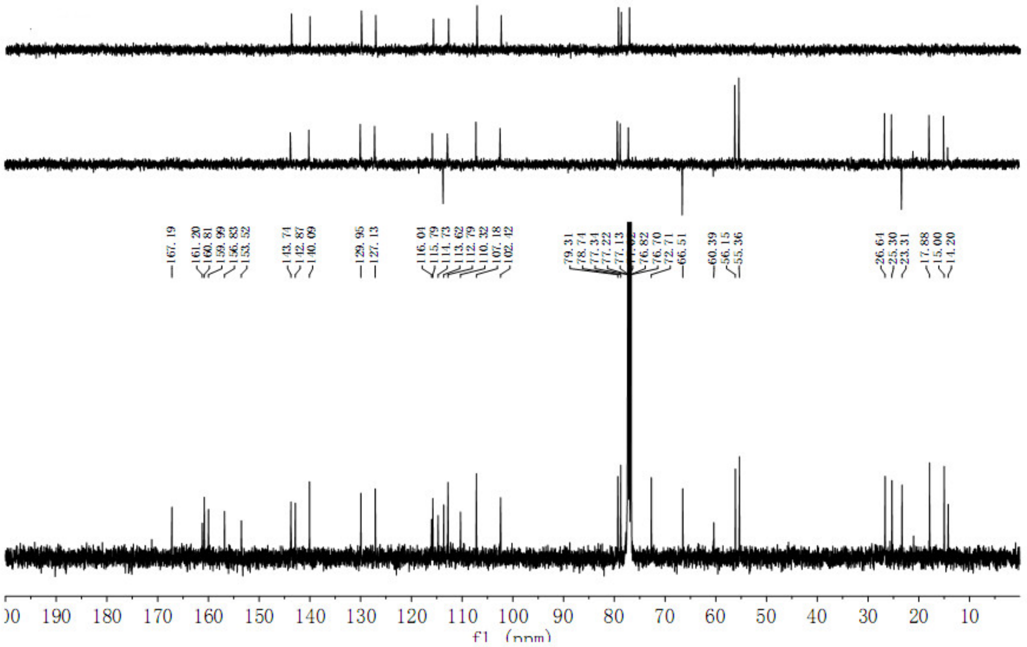

Figure S2. ^{13}C NMR and DEPT spectra of **1** in $CDCl_3$

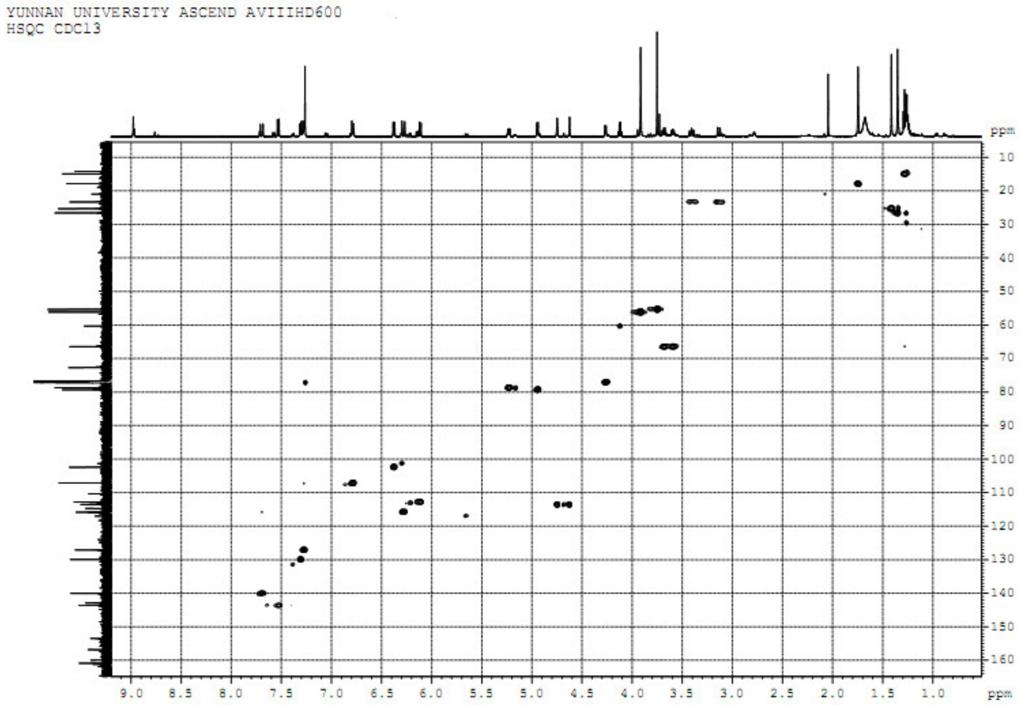

Figure S3. HSQC spectrum of **1** in $CDCl_3$

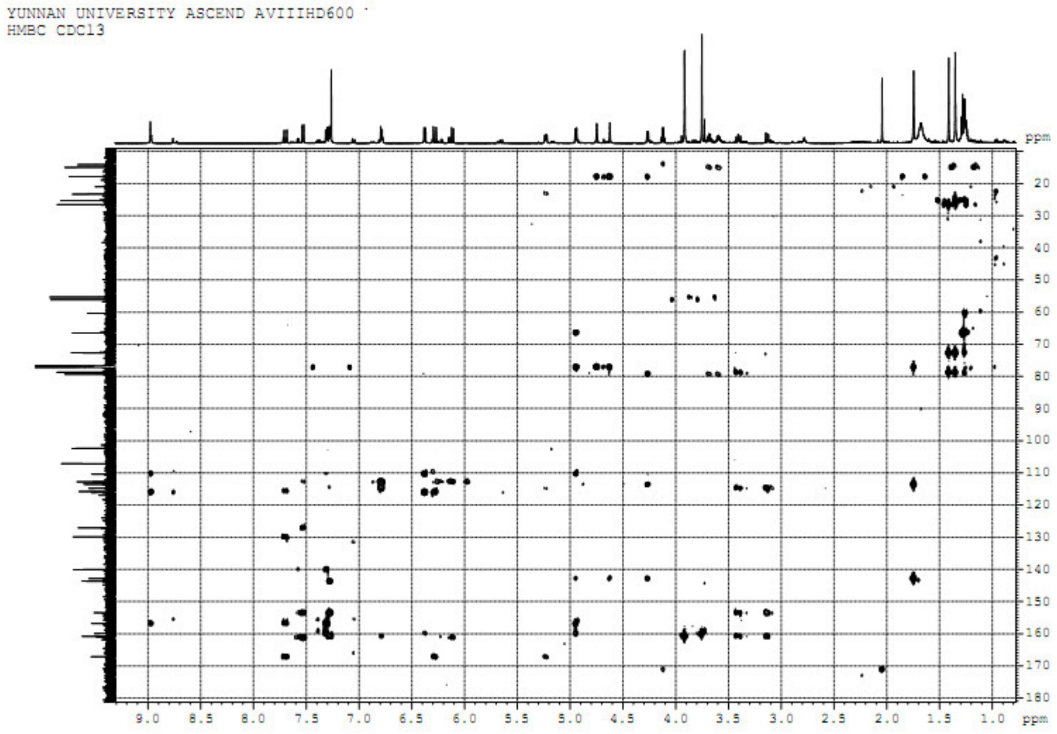

Figure S4. HMBC spectrum of **1** in $CDCl_3$

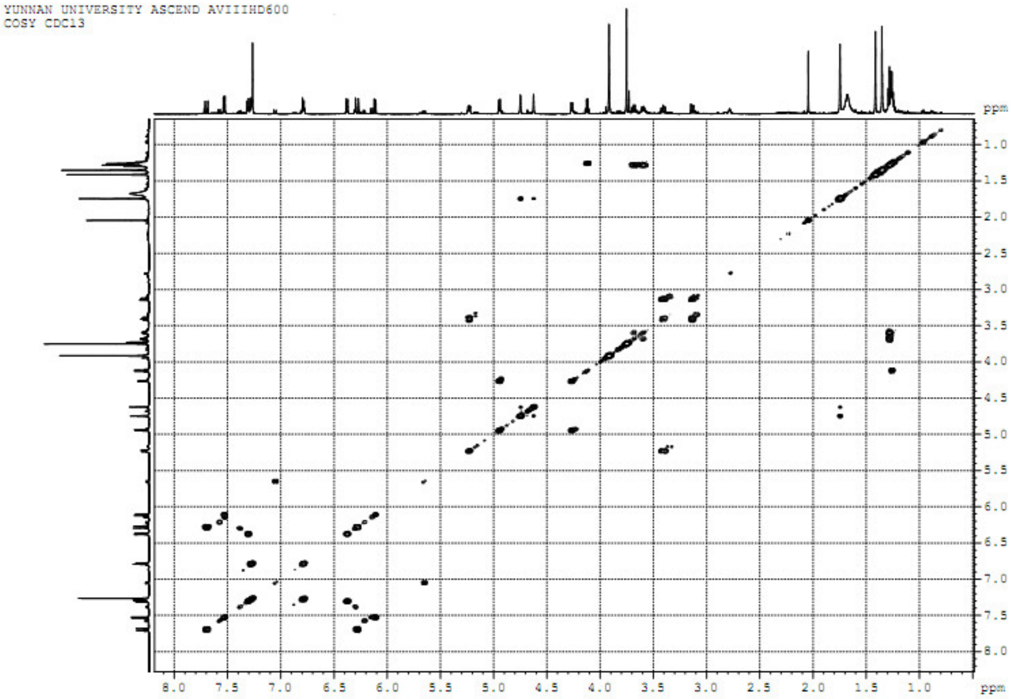

Figure S5. ^{1}H-^{1}H COSY spectrum of **1** in $CDCl_3$

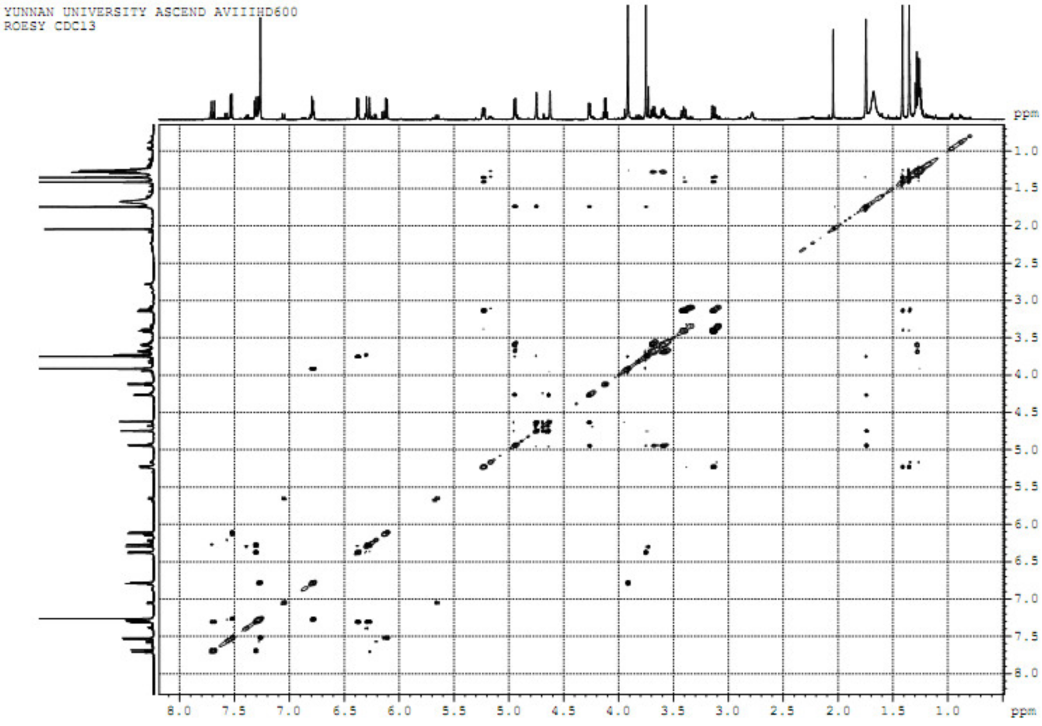

Figure S6. ROESY spectrum of **1** in $CDCl_3$

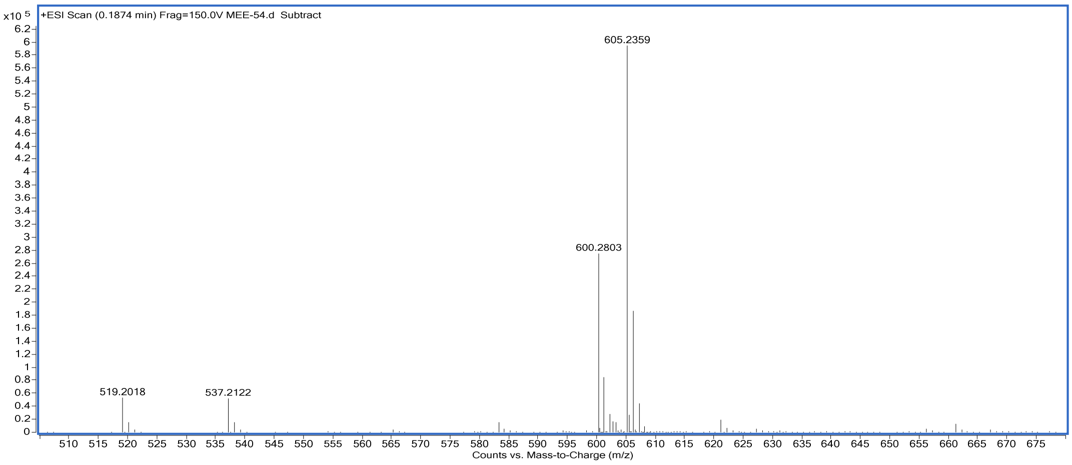

MS Formula Results: + Scan (0.1874 min)

m/z	Ion	Formula	Abundance
600.2803	(M+NH4)+	C32 H42 N O10	274790.1

Best	Formula (M)	Ion Formula	Score	Cross Score	Calc m/z	Diff (ppm)	Mass Match	Abund Match	Spacing Match
✓	C32 H38 O10	C32 H42 N O10	74.67		600.2803	0	100	53.4	49.54

m/z	Ion	Formula	Abundance
605.2359	(M+Na)+	C32 H38 Na O10	594872.8

Best	Formula (M)	Ion Formula	Score	Cross Score	Calc m/z	Diff (ppm)	Mass Match	Abund Match	Spacing Match
✓	C32 H38 O10	C32 H38 Na O10	97.29		605.2357	-0.39	99.82	92.86	97.56

Figure S7. HRESIMS spectrum of **1**

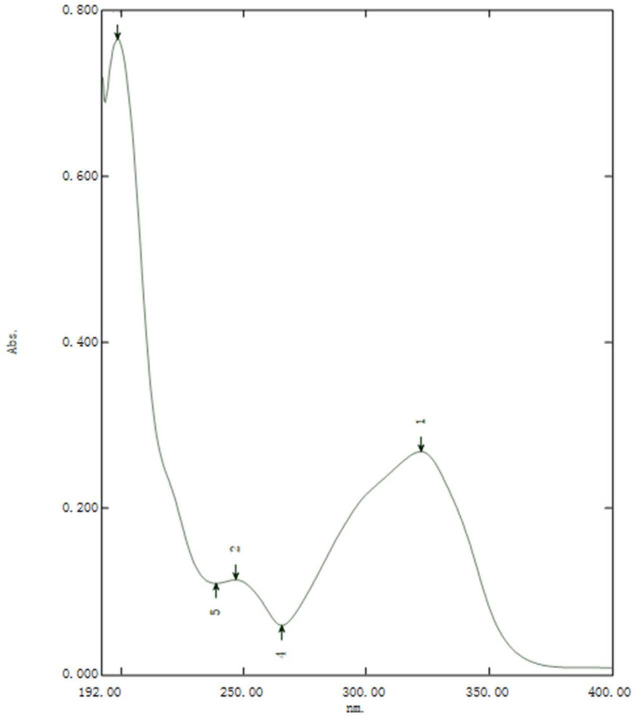

Figure S8. UV spectrum of **1**

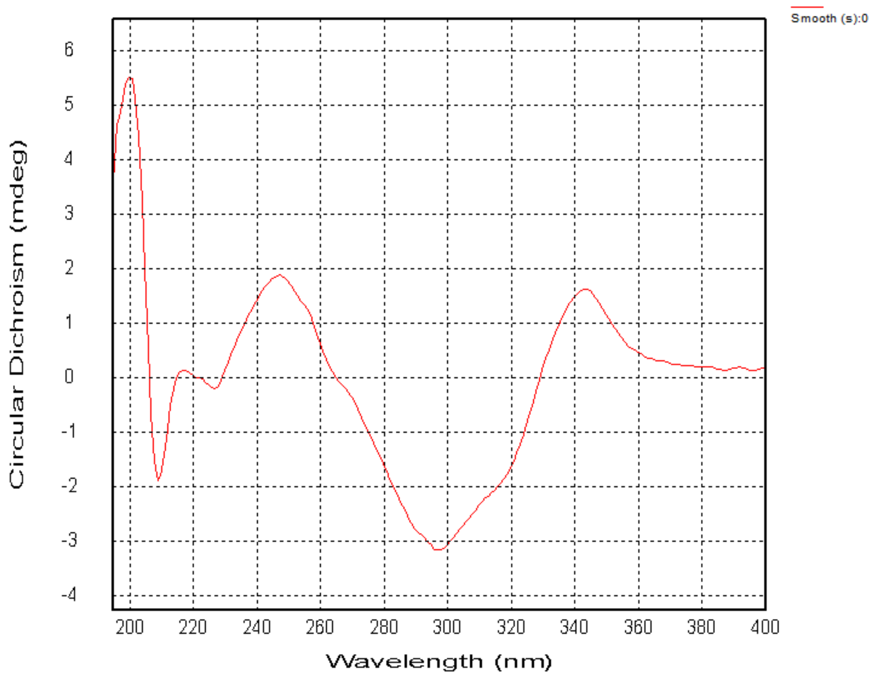

Figure S9. CD spectrum of **1**
